# Good Manufacturing Practice (GMP) Compliance for Phage Therapy Medicinal Products

**DOI:** 10.3389/fmicb.2020.01161

**Published:** 2020-06-04

**Authors:** Laurent Bretaudeau, Karine Tremblais, Françoise Aubrit, Marc Meichenin, Isabelle Arnaud

**Affiliations:** ^1^LB4Biotech Consulting, Nantes, France; ^2^Clean Cells, Boufféré, France

**Keywords:** phage, phage therapy, GMP manufacturing, process development, quality controls

## Abstract

Facing the emergence of difficult-to-treat bacterial infections, the perspective of using bacteriophages has re-gained interest in many countries. In terms of pharmaceutical classification in EU and United States, phages are considered as anti-infectious medicinal products and biological products, given the intended use and their live nature. During the production steps, the compliance with the Good Manufacturing Practice (GMP) represents the gold-standard to ensure the quality, safety and efficacy of medicinal products, either investigational or approved. In practice, the implementation of GMP rules for phage therapy medicinal products benefits from the long history of vaccine development. Accordingly, a well-structured strategy can be defined for each medicinal product, taking into account the specified indication (i.e., the target bacteria species, the infected site, the route of administration, the product composition). Based on the experience of different phage therapy medicinal products from the recent years, the most important requirements to achieve and claim GMP grade are reviewed here, including for genetically modified phages. Like all new medicinal products, the manufacturing of investigational phages incorporates significant challenges. However, the use of GMP-certified phages provides the best guarantee for the rigorous assessment of quality, safety and efficacy during the clinical development of phage medicinal products, thus appears as a key component for the successful development of phage therapy approaches.

## Regulatory Context, Manufacturing Requirements and Market-Access Perspectives

Phages are envisioned for a variety of uses including (1) the biocontrol of pathogenic bacteria in agriculture and food industries, (2) the modulation of dysbiotic flora, (3) the eradication of pathogenic bacteria infecting humans or animals. The scope of the present review is limited to the medical setting in human, when a therapeutic effect is needed against a clearly defined bacterial target (or a few defined targets simultaneously). This description actually matches the definitions of “medicinal products” in the European directive 2001/83/EC (European Commission, 2019a) and “drugs” by the Federal Food, Drug, and Cosmetic Act (FD&C Act) (US Code 21 §321) (United States Code, 2019). For clarity, the term “medicinal products” will be used in the rest of the present review.

However, independently from the classification as medicinal products in EU and United States, some phages are produced in different countries, notably Poland, Georgia, based on an historical use of phages and under local authorizations. There are at least two circumstances for the use of phages:

1. Phages prepared for a single patient and administrated to this patient [often, but not exclusively, as last resort approach under the umbrella of the article §37 from the declaration of Helsinki (World Medical Association, 2019)].
2. Phages widely available over the counter (i.e., without certainty to effectively impact the pathogenic strain from a patient).

Despite encouraging reports on case studies (Miȩdzybrodzki et al., 2018), this practical experience has not allowed so far to collect strongly structured data on safety, efficacy, pharmacokinetic and pharmacodynamic, that would support the improvement of the practice and the acceptability of phages as therapies in other countries.

In the recent years, the Belgian phage community has been very active in seeking options for implementing phage therapy with patient-specific phages. As a result, a monograph describing the production and characterization of phages suitable for magistral preparations was prepared. This monograph was endorsed by the Belgian health authority (FAMHP), and currently represents the most advanced regulatory framework in EU (Pirnay et al., 2018). Such magistral preparation framework allows to prepare patient-specific phages, under the responsibility of a medical doctor and a pharmacist. While this approach is fitted to treat individual cases (notably in last resort situation), its extension to clinical trials has not been engaged so far (Pelfrene et al., 2019). Such clinical trials with adaptative products are feasible, and needed to rationally characterize medicinal products and to support the industrial scale up that is required to reach the in-demand population.

With the classification of phages as medicinal products, direct requirements from the health agencies are that the products given to the patients (1) follow the gradual evaluation through clinical trials before obtaining a Market Authorization, and (2) are manufactured in compliance with the Good Manufacturing Practice (GMP) rules (European Commission, 2019b). The goal of GMP is to ensure the safety and efficacy of manufactured drugs, by relying on consistent manufacturing process and rigorous quality control (QC) program. In practice, the manufacturing process is controlled through the implementation of numerous tasks to document the compliance of all the parameters involved, notably: (i) the training and validation of the personnel, (ii) the qualification and the monitoring of the facility, the equipment, (iii) the qualification and controls on the raw material and consumables, (iv) the validation of the process and the QC methods, (v) the inspection by the relevant authorities.

When medicinal products are in development stages, there are a number of uncertainties, such as the potential impact of impurities or the effective dose required. Once clinical trials are engaged, the information on putative safety issues and efficacy can direct some evolutions of the manufacturing steps. In practice, this translates in an adaptative implementation of GMP, with increasing stringency along the development stages of the medicinal product.

The development of phages as medicinal products benefits from the long experience acquired in the field of vaccines for human and veterinary use. The manufacturing strategy of vaccines was inspiring for the consensus elaboration of the requirements for phage medicinal products (Pirnay et al., 2015). The general organization of production relies on the preparation of bacteria cell banks and phage stocks, that are controlled for their identity/purity/potency and used to develop and validate the manufacturing process. Once these steps are done, the manufacturing of GMP batches can be performed. For Genetically Modified Organism (GMO) -phages, some additional requirements are needed (see below).

Similar to most drugs, the goal of developing phage medicinal products is to reach a market authorization, so that they are available to patients. Gathering the clinical evidences to support the application for marketing authorization is thus a priority for phage medicinal products developers.

In the recent years, results from clinical trials and case reports have been reported (Furfaro et al., 2018). One of the conclusions is that the safety profile of phages is satisfactory. However, given the high specificity of interaction between a phage and a subset of bacteria, the capability of a phage medicinal product to target a patient-specific isolate is sometimes difficult to attain. In the case of *Propionibacterium acnes*, due to the low biodiversity of the bacteria, it is achievable to target almost all isolates with a drug containing 3–4 phages (Marinelli et al., 2012; Liu et al., 2015). For *Staphylococcus aureus*, it also appears feasible to cover most isolates with a combination of 3–4 phages, assembled in a single drug (Lehman et al., 2019). In contrast, the isolates from *Escherichia coli* (Sorin Bolocan et al., 2016; Jault et al., 2019) or *Acinetobacter baumanii* (Schooley et al., 2017) are so diverse that it is necessary to screen high numbers of phages in order to find active ones. These two situations result in different approaches for the pharmaceutical development of phage medicinal products: for the first cases, a fixed drug can be developed according to the standard pathway of medicinal products (i.e., one medicinal product for the whole patient population); for the latter the development requires to follow a precision medicine approach with medicinal products prepared on an individual basis (similarly to allergen preparations).

In the indications where a fixed drug is relevant, the design of appropriate clinical trials faces a limited number of questions: is the phage drug used alone or in combination with antibiotics? is the objective to show non-inferiority or to show superiority to the standard of care? In contrast, the design of clinical trials with different medicinal products given to a population of patients appears challenging in order to make any conclusion possible on efficacy. In the hypothesis where different phage medicinal products targeting the same bacteria in a specified indication could demonstrate efficacy, then it might become possible to grant a “class authorization” for these different phages, e.g., myoviridae against bacteria X. The regulatory framework to support such scheme remains to be elaborated. It is worth noting that existing regulatory frameworks (e.g., allergens, auto-vaccines) can be inspiring as analyzed by Fauconnier (2018). In terms of operations, this approach would need to maintain stocks available for large series of phages and to organize the use of the relevant ones for a given patient, possibly via extemporaneous preparation. Currently, the business model for this approach remains to be consolidated, as the costs of maintaining stocks and operational capacities for on-demand medicinal product manufacturing represent a heavy load.

## Considerations for Gmo Phage Manufacturing

In the past years, most of the phage therapy projects involved the use of natural lytic phages as antimicrobial therapeutics. However, the interest in engineering phages is growing due to limitations of natural lytic phages such as the competition or neutralization of phages within cocktails, the difficulties in isolating lytic bacteriophages for certain bacterial species (Nobrega et al., 2015), the development of bacterial resistance to phage infection or the limited access to bacterial targets imbedded in biofilms. In addition, the generation of intellectual property around engineered phages plays a major role in the expansion of this area. It is worth noting that phage engineering is envisioned not only to treat antibiotic-resistant infections but also to contemplate new applications such as microbiota edition, drug delivery or vaccines. These extensions exemplify the intense scientific interest of phages out of the classical antibacterial vision of phage therapy but will not be discussed further in the present review.

The types of modifications that are explored are widespread, and include notably:

1. The modification or addition of phage components responsible for host binding (Yoichi et al., 2005; Yosef et al., 2017).
2. The regulation of replication mechanisms (e.g., by creating a recombinant phage capable of delivering a gene coding for a small acid-soluble spore protein (SASP) which can be produced by the bacteria and inactivate its DNA by irreversible binding) (Fairhead, 2009).
3. The modification of temperate phages to become permanently lytic (Dedrick et al., 2019).
4. The improvement of the activity of phages against biofilms (e.g., by expression of a biofilm-degrading enzyme (dispersin B) which attacks the glycocalyx and matrix of the biofilm) (Lu and Collins, 2007).
5. The sensitization of bacteria to antibiotics and selective killing of antibiotic-resistant bacteria by genome edition (Bikard et al., 2014; Citorik et al., 2014; Yosef et al., 2015).
6. The use of anti-CRISPR mechanisms, to overcome the resistance of bacteria to the phages (Stanley and Maxwell, 2018).

According to the type of genetic engineering and the application, the resulting GMO phages can be subject to additional regulations compared to natural phages.

As a biological medicinal product and GMO developed for the European market, it must comply with Directive 2001/83/EC (European Commission, 2019a); Directive 2001/18/EC (Article 12.2) (Eur-Lex, 2019a) and Regulation (EC) 726/2004 (Articles 6.2 and 6.3) (Eur-Lex, 2019b). An environmental risk assessment (ERA) needs to compile the scientific information on the probability of transmission of the GMO from the patient to other persons, animals, plants or the environment, based on appropriate assays (European Medicines Agency, 2019a, b). In line with their engineering, such GMO phages are likely to be considered as Advanced Therapy Medicinal Products (ATMP) by the European Medicines Agency (EMA), and benefit from a centralized authorization procedure. However, inter-state differences are known to be wide for the implementation of the GMO regulations across the European Union, resulting in uncertainties for accessing market in some states that globally reject GMO. When initiating a development project for the European market, it is necessary to incorporate enough flexibility to overcome the technical requirements as well as the regulatory constraints.

For the US market, innovative medicinal products are subject to 21CFR312 (Food and Drug Administration, 2019d), under the Food & Drug Administration (FDA) authority. Currently activities involving natural phage are supervised by the Office of Vaccines Research and Review (OVRR). GMO are supervised by the Office of Tissues and Advanced Therapies (OTAT). These offices are components of the Center for Biologics Evaluation and Research (CBER) at the FDA, it is likely that they would both participate in the evaluation of phage GMO project. A number of guidelines are available to address some specific features of GMO: (1) Chemistry, Manufacturing and Control (CMC), (2) clinical trials, (3) dissemination (Food and Drug Administration, 2019a,b,c).

The literature on the use of GMO phages in human is limited, illustrating that this branch has not developed yet. In the recent case report of a *Mycobacterium abscessus* infection (Dedrick et al., 2019), a strategy of phage engineering was adopted to generate a recombinant phage with the appropriate selectivity and deletions intended to convert the starting temperate phage to a lytic one. While the data support the principle of employing a GMO as a treatment, it is rather surprising that the dissemination and the impact on the other bacteria were not addressed. It appears likely that the regulatory agencies will follow this topic.

Despite of the GMO-specific constraints, the current trend for developing GMO phages relies on two valuable properties: first, the introduction of genetic elements allows to increase the potency of GMO phages versus wild-type phages, second the intellectual property linked to the design of phages with innovative properties fits better with the business strategy of investors and pharma partners. It is likely that these advantages will continue to fuel the clinical development of GMO phages in the coming years.

## Lessons From Practical Experience

The manufacturing of biologics such as phage medicinal products (natural or GMO) incorporates a high number of steps and of quality controls. Like for any drug, one objective in manufacturing phage medicinal products is to maintain the quality-cost-delay tryptic at an optimal value, to favor the fastest development of the drug. During the PhagoBurn project we collected data on the production of 28 different phages. These specific data represent a set of confidential information under the consortium agreement of the project, thus cannot be disclosed. However, hereunder, several considerations based on the GMP requirements (European Commission, 2019b) are listed and should be kept in mind to ease new projects.

### Starting Materials

The bacteria strain used for the propagation of a phage of interest represent a key component. The principle is to establish bacteria cell banks that can be consistently used for both the development steps and the GMP steps. At minimum, 100 vials are prepared for each cell bank. The details for the characterization are given in Pirnay et al. (2015), and mostly focus on the identity and the purity. In practice, it is useful to keep reliable records of the history of the strain and the characterizations that are gradually performed (e.g., demonstration of the absence of toxins, antibiotic-resistance genes, detection of prophages). The same principles apply to each phage of interest: assembling a dataset on its history (isolation, number and conditions for the amplification rounds) and the characterization (host range, sequencing), is necessary to document the purity and the stability. These tasks need to be anticipated by the research labs when it is envisioned to move toward clinical trials.

### Materials (Reagents, Consumables)

Like any biological manufacturing projects, the reagents and consumables need to fulfil selection criteria (animal-component free medium, USP class VI consumables). The documentation provided by each manufacturer (i.e., the Certificates of Analysis, CoA), attests from its engagements (1) to perform the claimed sourcing and production process, (2) to release the raw materials based on satisfactory quality controls

In addition to this documentation, the medicinal product manufacturer has the responsibility to check that the raw materials have the appropriate properties. Thus, it is standard procedures to perform audits of the raw material manufacturers (on site or remotely), and to perform additional quality controls on each batch of raw materials, based on the intended use (e.g., growth of bacteria according to pre-established criteria for a culture medium).

### Primary Containers

The primary containers are in direct contact with the final product, for long periods. Thus, the specifications are even higher than for raw materials, to reduce the risk of unexpected contaminants (glass, rubber, endotoxins, sterility, integrity of the capping system). As phages are live products, there is no possibility to add sterilizing steps once the distribution in the primary containers is done. It is thus critical to have (1) a strategy of selection and control of providers to maximize the safety before using the primary containers, (2) the processes to minimize the risks of external contamination during the use of the primary containers. During the stability studies, the integrity of the containers is also monitored.

### Cleaning and Decontamination

Given the small size of phages and the high titers obtained *in vitro*, it is absolutely necessary to establish procedures for the cleaning and decontamination that are highly efficient to avoid any cross-contamination during the development or manufacturing steps. The validation of detergents and decontamination procedures relies on well-established principles (International Standard on Organization, 2019), and its adaptation to the specific settings of phage is straight forward.

### Process

The process refers to the consecutive steps that are necessary to produce the expected amount of phages, with a satisfactory level of quality. In practice, a first objective of the process development is to determine the optimal propagation conditions to obtain a high titer of phages, while maintaining the impurities such as cell debris to an acceptable level. This first step is identified as “upstream development” and relies mostly on varying some culture parameters: density of bacteria, multiplicity of infection, culture medium, supplements, duration, temperature, shaking.

Once the propagation conditions are optimized, the second objective is the obtention of a purification process that allows to remove the unwanted material (intact bacteria cells, cell wall-derived endotoxins (Gram-negative bacteria), bacterial DNA) and to recover replicative phages. This second step is named “downstream process” and relies mostly on performing a filtration of the crude harvest (0.2 μm), followed by filtration and/or chromatographic purification steps. At the end of the downstream process, the phages are stored in an appropriate conservation solution.

The upstream and downstream process optimization need to be performed by trained scientist, with the capability to envision and evaluate different scenarios. This task is pivotal, as it results in optimized and consistent yields and quality, and also in optimized duration of each step. This task is the basis for the training of the GMP operators, which fully justify the rigorous investigation of the production parameters. The purified phages that are collected during the process development are also of great importance for the initiation of formulation and stability studies as soon as the downstream process is stabilized.

During a manufacturing project, the number of experiments to be performed for the process development is highly dependent on the data available from the originating laboratory, and the quality requirements for the final products. For this purpose, the approach of Quality by Design (QbD) is a valuable tool aimed at gathering the information and organizing the whole production in a cost-effective and timely manner (ICH,2019a). In particular, it helps to industrialize the platform processes (amplification, purification tasks) and to operate efficiently with the quality control tasks.

The definition of the critical quality attributes (CQA) for phage medicinal products was detailed in Pirnay et al. (2015), with the contribution of a wide panel of phage experts. The content of this publication remains a fully valid backbone and additional dimensions on the process design were recently published (Mutti and Corsini, 2019).

### Quality Control Methods

The strategy of QC is designed to ensure the identity, purity, quality of each phage therapy medicinal product, thus represents a cornerstone of each project. The QC strategy has a large coverage, including the starting biological material from research labs, the master bacteria cell banks, the working bacteria cell banks, the master phage banks, the working phage banks, the intermediates in the production process (notably the drug substances, the drug products and the in-process controls) (Pirnay et al., 2015). For the QC of bacteria, six independent assays, covering the viability, identity and purity topics, are usually performed at the different levels to support the characterization of the bacteria as production substrates. For the QC of phages, a series of 20 assays is used to characterize the viability, identity, purity, chemistry at the different levels of the production. In addition, the raw materials are also subject to their direct QC release tests. Of course, increasing the number of phages translates in a significant extra-work in QC.

In terms of methodology, it is necessary to qualify and validate the quality control methods based on existing references (ICH Q2R1) (ICH,2019b). However, the quality control strategy also needs to employ state-of-art technologies that become available during the lifespan of a product. In the past years, next-generation sequencing (NGS) has emerged as a powerful tool to analyze phage and bacteria. So far, the implementation of this approach as GMP-compliant assay has proven to be difficult, mostly due to the absence of a solid validation framework. As shown in the workflow from Philipson et al. (2018), numerous steps are needed to describe precisely each phage by sequencing. Such workflow appears as “fit for purpose” since it provides a deep characterization of each phage, but its validation remains to be achieved to meet pharmaceutical standards (e.g., 21CFR part11 compliance). In terms of resources, the constant evaluation of contemporary methods versus established ones is also demanding and often under-considered but ultimately it is critical.

At the initial steps of PhagoBurn, we supported the idea to prepare the cocktail of different phages at the bedside during the clinical trial, to ensure that each phage was present at a precisely known titer. Finally, the decision to prepare the cocktail long before use proved to be deleterious. Indeed, it would have been necessary to quantify each phage strain in the medicinal product (i.e., the cocktail). Unfortunately, an appropriate method to quantify 12 phages independently is still missing. As a consequence, the characterization of the cocktail as a medicinal product appeared inadequate and possibly contributed to trial failure. This highlights the interdependence between the careful determination of the more appropriate formulation and the QC strategy, as it may impact the whole development program.

### Infrastructure

The requirements from the GMP guidelines are extremely clear about the design, maintenance and control of the manufacturing facility, as well as the equipment used for production (European Commission, 2019b). These requirements appeared to be fully appropriate for the manufacturing of phages (e.g., using a class A safety cabinet in a class B room, or using an isolator (closed class A) is mandatory for sterile medicinal products), however, the validation of the cleaning/decontamination required additional work.

### Pharmaceutical Responsibilities

From the manufacturing site, the primary contact with the regulatory agencies is the Qualified Person (QP), who is responsible for the initial authorization of the pharmaceutical site and its maintenance over the duration of projects (European Commission, 2019b). As phages represented innovative medicinal products, it was necessary to establish constructive relationships with the different agencies in Europe (France, Belgium, Switzerland) during PhagoBurn project, in order to find the best responses to the questions and challenges that were encountered.

Like any GMP manufacturing projects, the responsibilities between the sponsor and the subcontracting manufacturing site are explicitly described in the manufacturing contract.

### Quality Assurance

The Quality Assurance (QA) manager and its team holds a transversal role across the manufacturing site, as the quality system management allows to document all the tasks under their proper procedures: the training and validation of operators, the good execution of technical tasks, the mastery of the infrastructure, the management of deviations, the management of corrective actions and preventive actions (CAPA). This important contribution is described in the GMP guideline (European Commission, 2019b). A broader perspective of the role of the QA has been elaborated in the ICH Q10, which describes one comprehensive model for an effective pharmaceutical quality system that can be implemented throughout the different stages of a product lifecycle (ICH, 2020). In practice for the manufacturing of medicinal products, the ICH Q10 directly refers to the QA management from the GMP guideline.

### Team

In the field of phage therapy, the projects are at early clinical trial stages. In this industrially immature context, gathering the expert team (scientific, technical, pharmaceutical experts) to ensure GMP compliant manufacturing represents a challenge. The rapid overview of the organization in the previous paragraphs shows that each topic is supervised by a group leader and the tasks are performed by dedicated operators. Like any project involving multiple contributors, the project management needs to be coordinated by a project leader, who links the internal groups and the external partners (sponsor, sub-contractors).

In practice, it is key to maintain trained laboratory personnel involved at each level from upstream (USP)/downstream (DSP) process and analytical methods development up to GMP manufacturing and quality control of the medicinal product. Scientific and regulatory knowledges, good documentation practices and transfer between development and GMP teams are essential. Establishing and maintaining a GMP site require expertise and dedicated resources, that represent a significant and continuous investment.

The PhagoBurn project showed that GMP manufacturing of phages is achievable (Jault et al., 2019) and other phage manufacturing projects have already benefited from this experience.

The tasks of propagation and purification of phages that are incorporated in the final investigational medicinal product represent only a marginal amount of the global project workload. As detailed in the present review, most of the workload is allocated to preparative steps, that bring proofs in the ability to re-produce new batches of phages with the same levels of quantity and quality.

### Project Design

As a final and constant recommendation, we would like to invite the interested project leaders to start the elaboration of their project with the final product in mind, as the objective to reach. Indeed, the nature of the final product (type of phages, administration route, presence of several phages in a cocktail, titer of each phage, number of patients) has a strong impact on the development and manufacturing tasks.

## Conclusion – Perspectives

In the recent years, the phage therapy has re-emerged as a promising approach against pathogenic bacteria, notably the antibiotic resistant ones. One of the most attractive characteristics of phages consists in their high precision to target a bacteria subset, while antibiotics have a wider spectrum and provoke collateral damage on non-pathogenic microbiota.

The reports on individual cases with phage therapy have attracted a lot of attention, but they remain limited to a few patients without alternative therapeutic options [e.g., 15 patients from April 2013 to April 2018, at Queen Astrid Military Hospital, Brussels, Belgium (Djebara et al., 2019)]. These reports have the great value to (re-)open a fascinating therapeutic field, however, antibiotic resistance is such a massive threat to millions of humans that it is also necessary to consider the need to scale-up in an industrial setting.

When a large production is relevant, the implementation of GMP is fully appropriate. However, when exotic phages are necessary for a limited number of patients, it might be wise to define a less stringent format and the Belgian monograph represents an interesting step in this direction.

With the recent and forthcoming clinical observations, evolutions of the regulatory framework will be necessary to better describe and exploit the potential of phages, thus paving the way for future availability of phage medicinal products.

## Author Contributions

LB drafted and wrote the manuscript. KT and MM contributed on quality controls. FA contributed on manufacturing process. IA contributed on regulatory compliance. KT, MM, FA, and IA reviewed the manuscript.

## Conflict of Interest

LB is a former employee at Clean Cells. LB has performed consulting activities for Clean Cells. KT, FA, and IA are employees at Clean Cells. MM is co-founder and shareholder of Clean Cells.
